# Cellular Gene Expression during Hepatitis C Virus Replication as Revealed by Ribosome Profiling

**DOI:** 10.3390/ijms20061321

**Published:** 2019-03-15

**Authors:** Gesche K. Gerresheim, Jochen Bathke, Audrey M. Michel, Dmitri E. Andreev, Lyudmila A. Shalamova, Oliver Rossbach, Pan Hu, Dieter Glebe, Markus Fricke, Manja Marz, Alexander Goesmann, Stephen J. Kiniry, Pavel V. Baranov, Ivan N. Shatsky, Michael Niepmann

**Affiliations:** 1 Institute of Biochemistry, Medical Faculty, Justus-Liebig-University, Friedrichstrasse 24, 35392 Giessen, Germany; gesche.gerresheim@gmx.de (G.K.G.); cannyhp@126.com (P.H.); 2 Bioinformatics and Systems Biology, Faculty of Biology and Chemistry, Justus-Liebig-University, 35392 Giessen, Germany; jochen.bathke@agrar.uni-giessen.de (J.B.); Alexander.Goesmann@computational.bio.uni-giessen.de (A.G.); 3 School of Biochemistry and Cell Biology, University College Cork, Cork T12 XF62, Ireland; audreymannion@gmail.com (A.M.M.); 114224403@umail.ucc.ie (S.J.K.); p.baranov@ucc.ie (P.V.B.); 4 Belozersky Institute of Physico-Chemical Biology, Lomonosov Moscow State University, Moscow 119234, Russia; cycloheximide@yandex.ru (D.E.A.); ivanshatskyster@gmail.com (I.N.S.); 5 Institute for Virology, Faculty of Veterinary Medicine, Justus-Liebig University, 356392 Giessen, Germany; ludmilashalamova@gmail.com; 6 Inst. of Biochemistry, Faculty of Biology and Chemistry, Justus-Liebig-University, 35392 Giessen, Germany; Oliver.Rossbach@chemie.bio.uni-giessen.de; 7 Inst. of Medical Virology, Justus-Liebig-University, National Reference Center for Hepatitis B and D Viruses, Giessen, and German Center for Infection Research (DZIF), partner site Giessen, 35392 Giessen, Germany; Dieter.Glebe@viro.med.uni-giessen.de; 8 Genevention GmbH, 37079 Göttingen, Germany; markus.fricke2@googlemail.com; 9 RNA Bioinformatics and High Throughput Analysis, Faculty of Mathematics and Computer Science, Friedrich Schiller University Jena, 07743 Jena, Germany; manja@uni-jena.de

**Keywords:** HCV, HCC, hepatocellular carcinoma, liver cancer, Warburg effect, ER stress, ribosome profiling, Riboseq, respiratory chain, mitochondria

## Abstract

Background: Hepatitis C virus (HCV) infects human liver hepatocytes, often leading to liver cirrhosis and hepatocellular carcinoma (HCC). It is believed that chronic infection alters host gene expression and favors HCC development. In particular, HCV replication in Endoplasmic Reticulum (ER) derived membranes induces chronic ER stress. How HCV replication affects host mRNA translation and transcription at a genome wide level is not yet known. Methods: We used Riboseq (Ribosome Profiling) to analyze transcriptome and translatome changes in the Huh-7.5 hepatocarcinoma cell line replicating HCV for 6 days. Results: Established viral replication does not cause global changes in host gene expression—only around 30 genes are significantly differentially expressed. Upregulated genes are related to ER stress and HCV replication, and several regulated genes are known to be involved in HCC development. Some mRNAs (*PPP1R15A*/*GADD34*, *DDIT3*/*CHOP*, and *TRIB3*) may be subject to upstream open reading frame (uORF) mediated translation control. Transcriptional downregulation mainly affects mitochondrial respiratory chain complex core subunit genes. Conclusion: After establishing HCV replication, the lack of global changes in cellular gene expression indicates an adaptation to chronic infection, while the downregulation of mitochondrial respiratory chain genes indicates how a virus may further contribute to cancer cell-like metabolic reprogramming (“Warburg effect”) even in the hepatocellular carcinoma cells used here.

## 1. Introduction

Hepatitis C Virus (HCV) causes liver disease by preferentially infecting human liver hepatocytes [1]. Acute HCV infections remain asymptomatic in most cases and the virus is cleared, whereas only in rare cases, patients develop acute life-threatening liver disease. However, in about 60–80% of infected persons the virus is not eliminated but continues to chronically replicate in the liver. Worldwide, about 71 million people are chronic carriers of HCV, of which about 15–30% develop liver cirrhosis within 20 years [2,3], which finally may lead to hepatocellular carcinoma (HCC). Among those chronic HCV patients who underwent anti-HCV treatment, the incidence of HCC is about 1–2% per year, but with increasing age the incidence can be as high as 10% per year [4,5]. Although powerful direct-acting antivirals (DAAs) have now been available for years, the high costs of treating chronic HCV infection by DAAs, as well as the emergence of resistant-virus isolates remain serious problems of disease control and underline the persisting need for a better understanding of cellular changes during HCV infection and disease development [6,7]. In particular, HCC recurs with a very high incidence even after successful clearance of HCV [4]. 

**Figure 1:**
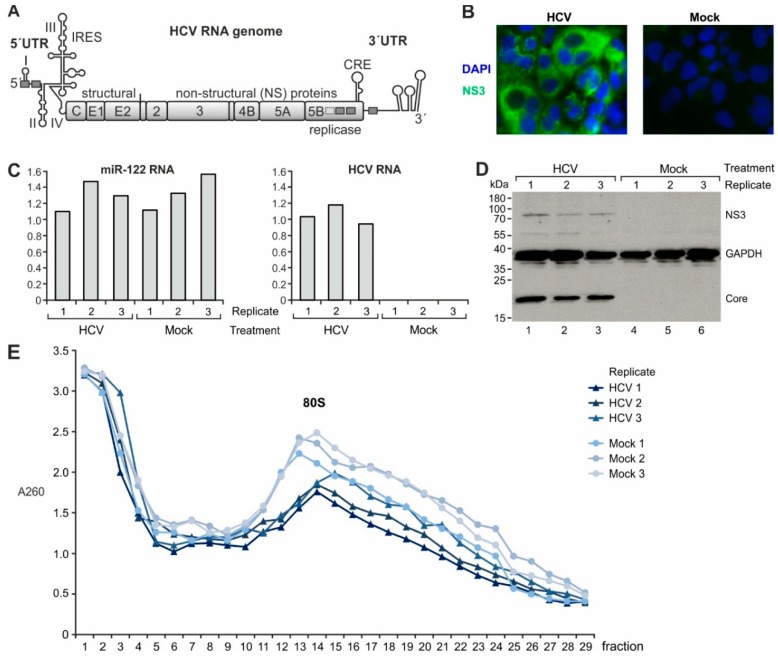
Hepatitis C Virus (HCV) replication in Huh-7.5 cells. (**A**) Full-length HCV genomes were transfected into Huh-7.5 cells. Six days after transfection, replication of HCV in the cells was assessed by detection of HCV NS3 protein (200-fold magnification) (**B**), HCV genomic RNA and miR-122 (**C**) as well as HCV NS3 and Core proteins by Western Blot. GAPDH (glycerol-3-phosphate dehydrogenase) was analyzed as loading control (**D**). (**E**) Cytoplasmic cell extracts were subjected to sucrose gradient centrifugation in order to enrich 80S ribosomes.

HCV belongs to the family *Flaviviridae* and has a positive-sense single-stranded RNA genome of about 9600 nucleotides (Figure 1A) [8]. The infectious virus comes as an enveloped lipoviral particle that contains viral proteins as well as cellular lipids and proteins [1,9,10]. The hepatotropism of HCV is in part due to a variety of receptors bound by the virus [1]. After infection of the cell, the HCV RNA genome is translated in the cytoplasm by virtue of an internal ribosome entry site (IRES) element located in its 5′ leader (for a review see [11]). Viral proteins which are processed from the precursor polyprotein then induce the formation of double membrane vesicles that derive from the endoplasmic reticulum (ER) and form a so-called membranous web which provides a protected environment for replication of the viral RNA [10,12]. The liver-specific microRNA-122 (miR-122) is involved in enhancing replication, translation, and stability of the HCV genome [13,14,15] and by that considerably contributes to the hepatotropism of HCV. 

The highly conserved RNA secondary structure and sequence *cis*-elements involved in genome replication are located near the genome ends as well as in the protein coding region [8,11,16,17,18]. The infecting viral plus-strand RNA genome is copied to produce an antigenome minus strand by virtue of the replication complex including the viral non-structural (NS) proteins NS3-NS5B. From this minus strand, progeny plus strands are then produced in large excess [16]. After that, translation from the progeny plus strand genomes produces large amounts of viral proteins [16,19], and finally the viral progeny genomes are encapsidated into newly assembling viruses [9,10,20,21]. HCV particle assembly not only involves viral proteins but also cellular proteins, in particular some of which are involved in the metabolism of very low density lipoprotein (VLDL) and low density lipoprotein (LDL) particles that are routinely synthesized by the hepatocytes to deliver various lipids to other organs. Proteins involved are in particular the apolipoproteins ApoE, ApoAI, ApoCII, and ApoB [9]. 

Upon HCV infection, the expression of cellular genes is subject to various changes. HCV replication in the ER-derived membranes causes ER stress [22]. Several mechanisms of the innate and adaptive immune system are activated [23,24,25]. However, the virus counteracts many of these steps by interactions of its proteins with components of the cellular innate immune system, often leading to failure of the innate immune response in HCV infection [23,24]. Also, the adaptive immune response, e.g., by cytotoxic T cells, often fails [23,24]. Moreover, fast generation of HCV escape mutants due to its error-prone replicase is among the reasons that the adaptive immune response is unable to keep pace with the molecular evolution of HCV [25]. 

Development of hepatocellular carcinoma as a consequence of chronic HCV infection is yet far from being completely understood [26,27,28]. Cellular changes that may contribute to cancer development include escape from immune responses as described above, evasion from the control of growth and tumor suppressors, escape from apoptosis, genome instability and mutation, induction of angiogenesis, proliferative signaling as well as metabolic reprogramming [28,29,30] and the deregulation of cellular energetics. Mitochondrial bioenergetic imbalance has been shown to be due to calcium ion influx that is caused by the ER modifications at ER-mitochondrial fusion regions induced by HCV proteins, without providing a direct link to mitochondrial gene expression [31,32]. In addition, HCV core protein was shown to contribute to mitochondrial damage by impairing mitophagy [33]. Both mechanisms (and others) result in enhanced oxidative stress in the mitochondria and by that contribute to carcinogenesis. 

In this study, we aimed to detect changes in cellular gene expression due to transcriptional as well as translational control after ongoing HCV replication was fully established in the cells. To this end, we analyzed cellular expression changes 6 days after the onset of HCV infection. Even though the use of Huh-7.5 hepatocarcinoma cells in our study already comes with the limitation that we could not observe a full change from primary liver hepatocyctes to HCC cells, our results show the upregulation of genes involved in ER stress, autophagy, metabolic reprogramming and further contribution to HCC development. Translation of some of these mRNAs may involve regulatory upstream open reading frames (uORFs). Moreover, downregulation of several mitochondrial respiratory chain key genes indicates a downregulation of oxidative phosphorylation, a novel possible contribution to the development of the Warburg effect that enhances metabolite flux in cancer cells despite ongoing oxidative phosphorylation. 

## 2. Results and Discussion

### 2.1. Gene Expression Analysis in Cells Replicating HCV

We have chosen the popular model of Huh-7.5 hepatocarcinoma cell line which is fully capable to support all stages of the viral life cycle. The cells were transfected with full-length HCV genomes (Figure 1A) and allowed to replicate and spread the virus for 6 days. This protocol was chosen in order to approach conditions of chronic HCV replication in cells. A full replication cycle of HCV takes less than 24 h [34]. Therefore, after 6 days all cells are infected by the virus (Figure 1B), the HCV genomes have undergone multiple rounds of replication, and the situation in the cells can be regarded to mimic fully established ongoing HCV replication that resembles chronic HCV infection. Cellular miR-122 concentrations were similar in all HCV and mock transfected replicates (Figure 1C, left panel), while HCV RNA (Figure 1C, right panel) and HCV proteins (Figure 1D) were detected only in HCV-transfected cells. 

We then analyzed both the transcriptional as well as the translational regulation of mRNAs in the HCV replicating cells. Therefore, we isolated total RNA from the cells to analyze transcript abundance by next generation sequencing (NGS). In parallel, we isolated the RNA fragments occupied by ribosomes using the ribosome profiling method [35,36]. In addition to the Ingolia protocol [36], we enriched 80S ribosomes by sucrose gradient centrifugation (Figure 1E, “80S”) to ensure that we selectively capture RNA that actually is occupied by ribosomes [37], in contrast to other RNA that may have been unspecifically pelleted in aggregates. 

**Figure 2:**
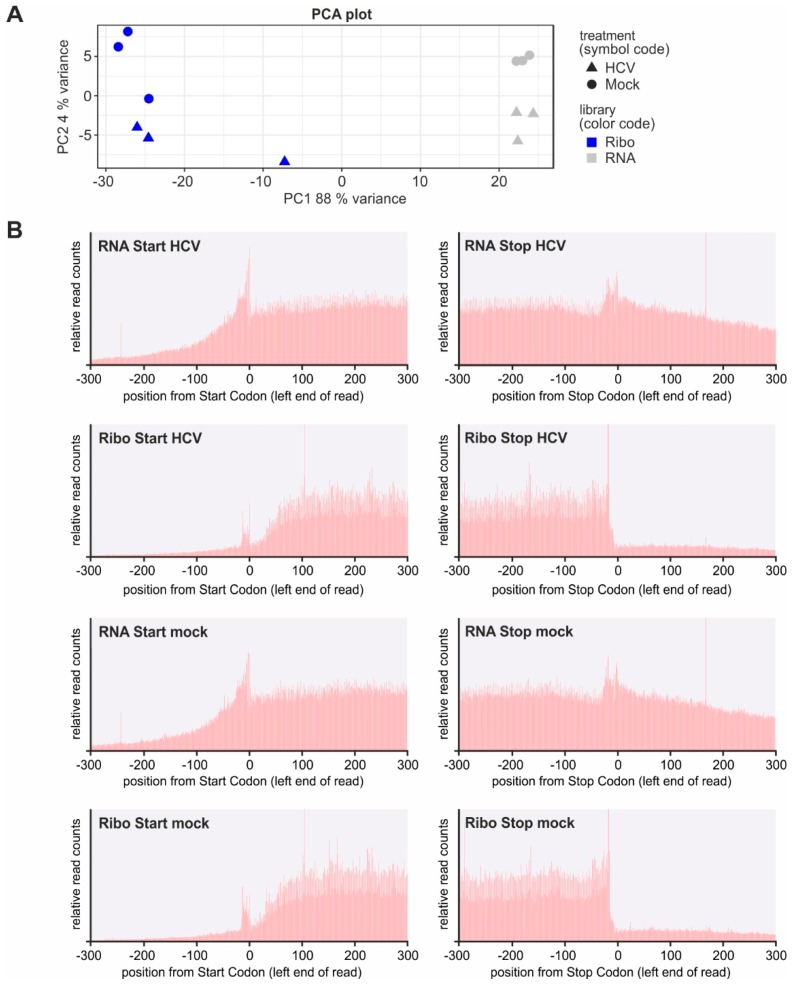
Quality controls of transcriptome and ribosome profiling analyses. (**A**) Principal component analysis (PCA) of gene expression in transcriptome and ribosome profiling replicates. (**B**) Metagene analyses of transcriptome reads (“RNA”) and ribosome profiling reads (“Ribo”) in HCV-replicating cells and non-transfected cells (“Mock”) around the Start and Stop codons of cellular mRNAs.

Overall analysis of cellular gene expression by principal component analysis (PCA; Figure 2A) shows that mRNA expression in the triplicates of HCV replicating cells closely clusters separately from the cluster of mock-transfected cells (“RNA”, right). Reads representing ribosome occupancy (“Ribo”, left) largely differ from those of mRNA abundance, and also here HCV replicating cells differ from mock-transfected cells. In the ribosome profiling analyses, one of the replicates of each treatment is somewhat separate from the other two in the PCA plot. Therefore, in further analyses (see below) we have tested differential gene expression by comparing each of the three replicates of one treatment (e.g., HCV) against each replicate of the other treatment (mock) in all possible crosswise combinations. In these analyses, the results were very similar also when each one of the replicates was omitted from the analysis (not shown). Due to the robustness of these results we conclude that downstream gene expression analysis yields a reliable representation of the differences in gene expression and ribosome occupancy between the different treatments. 

Metagene analysis of reads representing all RNAs in the cell (Figure 2B, panels “RNA”) shows that reads are distributed evenly over coding regions of mRNAs, and 3´UTRs (right panels) are longer than 5´UTRs (left panels) on average. Ribosome occupancy (panels “Ribo”) shows a small peak at the start site, which may, however, in part be due to sequence biases since peaks in the RNAseq profiles coincide with the start. Moreover, a “ramp” of ribosomes queuing in the first 100 nucleotides of the coding sequence is observed, which may be due to ribosomes queuing downstream of the start codon as to be expected [38]. Then follows a rather even distribution of ribosomes over the remaining coding sequence and a sharp decrease in read numbers downstream of the stop codon. All these metagene analysis results are as expected and thus serve as a quality control. The results also show that HCV replication does not drastically change the relative distribution of ribosomes on the cellular mRNAs. 

### 2.2. Established Viral Replication Does Not Cause Global Changes in Host Gene Expression

**Figure 3:**
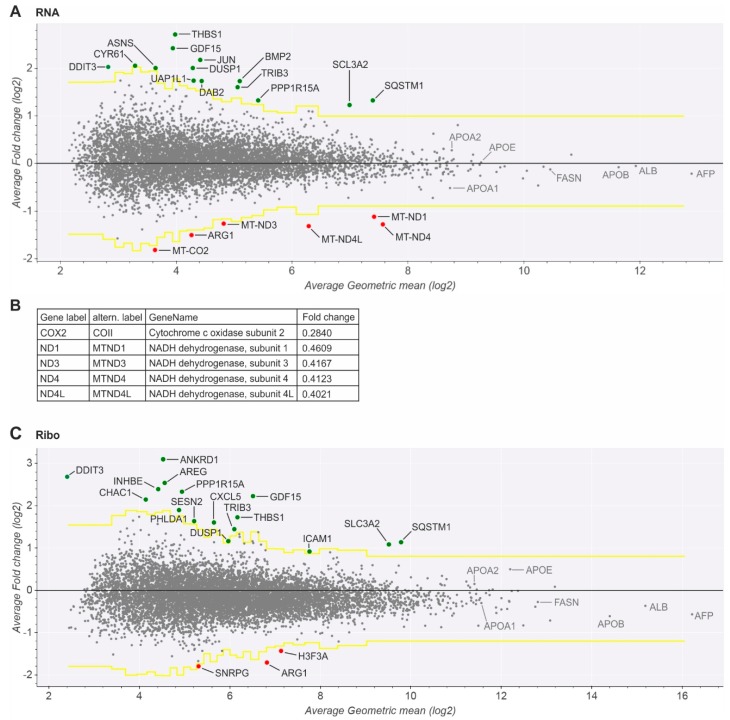
Gene expression in HCV-transfected versus mock-transfected cells 6 days after transfection, analyzed with the Differential plot in Trips-Viz [39]. (**A**) Transcriptome analysis (“RNA”). The x-axis denotes the log(2) of geometric mean read counts in complete mRNAs (minimum number of reads = 3, window size 25–40 bp) normalized by mapped reads, the y-axis the average log(2)-fold change (LFC) of expression. The black line indicates LFC = 0. The yellow lines indicate the significance threshold. Significantly upregulated genes are shown in green, downregulated genes in red. *DDIT3* was under the read count threshold but is noted in the plot. (**B**) Downregulation values of mitochondrial genes in detail. (**C**) Ribosome profiling analysis (“Ribo”). Details are as in (**A**), but reads were counted in the coding sequence only.

Comprehensive analyses of changes in cellular transcription in HCV replicating Huh-7.5 cells compared to non-infected cells show that the average transcription level of most mRNAs does not significantly change during HCV replication compared to non-infected cells. Only around 30 genes are differentially expressed. Fourteen mRNAs are significantly upregulated on the transcriptional level (Figure 3A), while even only six mRNAs are significantly downregulated. Analysis of ribosome occupancy of the cells by ribosome profiling shows upregulation of only very few mRNAs (Figure 3C). Sixteen RNAs are significantly upregulated and only three downregulated, many of them overlapping with those upregulated already on the transcriptional level. In conclusion, after establishing HCV replication in the cells, chronic HCV replication avoids global changes in the transcriptome and translatome. 

Only very few genes were found to be transcriptionally downregulated (Figure 3A). One of them is arginase 1 (*ARG1*), an important enzyme of the urea cycle in hepatocytes. Its activity of releasing urea from arginine is key to controlling the overall release of nitrogen from the body. It may be tempting to speculate if the downregulation of arginase may contribute to saving amino acids for HCV replication and tumor growth. Two other genes that are strongly downregulated on the translational level are *H3F3A* (H3 Histone Family Member 3A) and *SNRPG* (Small Nuclear Ribonucleoprotein Polypeptide G) (Figure 3C). 

Some well-known genes are highly expressed. Alpha-fetoprotein (*AFP*) is preferentially expressed in fetal and neonatal but not in adult liver. However, expression of *AFP* is reactivated during adult liver regeneration and hepatocarcinogenesis [40]. Thus, the high *AFP* expression observed here can be regarded as a tumor cell marker of the Huh-7.5 hepatocarcinoma cells. In contrast, several highly expressed genes are characteristic for liver cells, like serum albumin (*ALB*). Apolipoprotein B (*ApoB*) is expressed in liver cells for the routine production of VLDL and LDL particles that serve to transport lipids to the body´s periphery [41]. Other typical liver proteins involved in lipid metabolism like ApoA1, ApoA2, and fatty acid synthase (FASN) (Figure 3A, C) but also *ApoC1*, *ApoC2*, *ApoC3,* and *ApoM* (not shown) are expressed in the cells, indicating that the Huh-7.5 hepatocarcinoma cells retain a hepatocyte-like metabolic state, while their expression levels did essentially not change upon HCV replication. Also *ApoE*, which is involved in HCV particle assembly [21,42], is highly expressed in the Huh-7.5 cells. Some genes related to ER stress and hepatocellular carcinogenesis are upregulated (see below). However, we did not find significant differential expression of innate immune response genes, interferon-stimulated genes (*ISGs*) or changes in mTOR dependent translation. This suggests that HCV successfully counteracts persistent changes in gene expression of cellular defense genes. 

### 2.3. Key Mitochondrial Respiratory Chain Genes Are Downregulated

The other genes that are significantly downregulated on the transcriptional level code for subunits of the mitochondrial respiratory chain complex I (NADH-ubiquinone oxidoreductase) and complex IV (cytochrome c oxidase) (Figure 3A,B), while translational regulation of mitochondrial genes was essentially not captured by ribosome profiling due to the enrichment of cytoplasmic 80S ribosomes during sucrose gradient centrifugation. *MT-ND1*, *MT-ND3*, *MT-ND4*, and *MT-ND4L* constitute core subunits of complex I which are located directly within the inner mitochondrial membrane and are involved in the enzymatic activity of the complex [43,44]. Similarly, *MT-CO2* is a catalytically essential core subunit of complex IV, and also this subunit is located directly within the inner mitochondrial membrane [45]. Since these highly hydrophobic membrane proteins are essential components of the mitochondrial redox metabolism, they are encoded by mitochondrial genomes but not by nuclear genes to allow for short regulatory gene expression circuits [46], and their codon composition is markedly different from that of average nuclear genes [47]. 

The early downregulation of key mitochondrial respiratory chain genes may further contribute to the “Warburg effect” in the tumor cells [29,30,48,49]. The Warburg effect, also called “aerobic glycolysis”, means that in tumor cells the metabolite flux through the glycolysis and pentose phosphate pathways is strongly increased, while mitochondrial functions including oxidative phosphorylation are still required [29,30,48,49]. This adaptation is thought to be established to provide more metabolites for tumor cell growth, while this idea appears somewhat inconsistent with the high release of lactate by these cells. However, some reports have linked the downregulation of oxidative phosphorylation in mitochondria to the decreased expression of the catalytic β subunit of the F_1_ ATPase protein [50,51]. This could mean that the downregulation of oxidative phosphorylation and the upregulation of glycolysis may also be an adaptation to low oxygen supply in fast growing tumors lacking sufficient neovascularization. Moreover, mitochondrial bioenergetic imbalance during HCV infection has also been attributed to calcium ion influx that is caused by the ER modifications at ER-mitochondrial fusion regions induced by HCV proteins, without providing a direct link to mitochondrial gene expression [31,32], and HCV core protein was shown to contribute to mitochondrial damage by impairing mitophagy [33]. Our results shown here support the idea that downregulation of mitochondrial respiratory chain activity upon active HCV replication in the cells may further contribute to the Warburg effect, even though the Huh-7.5 cell line used here already is a hepatocarcinoma cell line. The changes of expression of these candidate genes can be evaluated in future studies in more physiological models of HCV infection (e.g., in primary hepatocyte cultures).

### 2.4. Upregulated Genes Are Related to ER Stress, HCV Replication, and Hepatocarcinogenesis

A limited number of genes is upregulated significantly (Figure 3A,C). The 22 upregulated genes include one (*DAB2*/*DOC-2*, clathrin adaptor protein) which is involved in a variety of signaling processes related to endocytosis [52], and 10 genes are known to be upregulated in response to ER stress (*ANKRD1*, *ASNS*, *BMP2*, *DDIT3*, *GDF15*, *INHBE*, *PPP1R15A*, *SESN2*, *SLC3A2*, and *TRIB3*). The latter may be due to the heavy changes that HCV replication applies to the ER in order to organize its replication [9,10,12]. 

*ANKRD1* (Ankyrin Repeat Domain 1) was shown to be upregulated during HCV infection by HCV NS5A protein and positively regulates HCV Entry [53]. *ASNS* (asparagine synthetase, glutamine-hydrolyzing) is known to be transcriptionally upregulated by ER stress or conditions of amino acid starvation [54]. *BMP2* (*BDA2*, bone morphogenetic protein 2) is involved in the induction of osteoblast differentiation [55]. *DDIT3* (*CHOP*; DNA Damage Inducible Transcript 3) is implicated in the regulation of autophagy and apoptosis [56]. *GDF15* (growth differentiation factor 15) is a member of the transforming growth factor-β cytokine superfamily. *GDF15* was induced in hepatocarcinoma cells by HCV and resulted in increased DNA synthesis, promoted cell proliferation, and enhanced invasiveness of the cells [57]. The beta E subunit of the transforming growth factor-β (TGF-β) superfamily members inhibin or activin (INHBE) is known to be upregulated as a transcriptional target gene of *ATF4* [58]. *INHBE* mRNA is predominantly expressed in liver, and it may inhibit cellular proliferation and growth [59]. Thus, in contrast to other regulated genes *INHBE* may counteract the induction of cell growth in the HCV infected cell due to the induced ER stress. *SESN2* (Sestrin 2) is a metabolic regulator which plays a role in protection against oxidative and genotoxic stresses. Like *SQSTM1* (see below), it was shown to induce *Nrf2*-dependent metabolic reprogramming by inducing the expression of genes for antioxidant enzymes [60]. Also the amino acid cotransporter SLC3A2 has been shown to be induced during ER stress [61].

Two of the above genes that are upregulated by ER stress (*PPP1R15A* and *TRIB3*) as well as three other genes (*CHAC1*, *DUSP1*, and *PHLDA1*) are also involved in the regulation of signaling pathways. CHAC1 (Glutathione Gamma-Glutamylcyclotransferase 1) degrades glutathione and thereby is involved in regulation of the oxidative balance in the cell. Glutathione depletion is an important factor for the initiation of apoptosis. Therefore, CHAC1 acts as a pro-apoptotic component of the unfolded protein response pathway [62]. DUSP1 (CL100, dual specificity phosphatase 1) is involved in dephosphorylation of mitogen-activated protein (MAP) kinases [63]. *PHLDA1* (Pleckstrin Homology Like Domain Family A Member 1) is induced by ER stress and may have tissue-specific roles in either the inhibition or elevation of cancer progression and metastasis [64]. 

PPP1R15A (GADD34, protein phosphatase 1 regulatory subunit 15A) is a stress-inducible subunit of the heterodimeric eIF2α holophosphatase that antagonizes the action of eIF2α kinases [65]. During HCV infection *PPP1R15A* expression is thought to counteract the stress-induced phosphorylation of eIF2. Upon infection of primary human hepatocytes with HCV, *PPP1R15A* transcription was shown to be induced, and the stress-induced phosphorylation of eIF2 and the induction of *PPP1R15A* led to oscillating activity of these both proteins [22]. 

TRIB3 (C20orf97, tribbles pseudokinase 3) is a regulator protein that has been implicated in the control of stress response, cell viability, and metabolic processes [66]. Under ER stress conditions, *TRIB3* is known to promote apoptosis by negatively regulating the Akt signaling pathway. While *TRIB3* mRNA and protein expression levels increase in HCV-infected cells, the *TRIB3*-*Akt* signaling pathway is disrupted by the HCV NS3 protein [67]. Thus, HCV modulates the *TRIB3*-*Akt* signaling pathway to prevent apoptosis and establish persistent viral infection.

One of the above genes which are upregulated during ER stress (*GDF15*) and seven others (*AREG*, *CXCL5*, *CYR61*, *JUN*, *THBS1*, *UAP1L1*, and *SQSTM1*) are known to be involved in cancer development including particularly also HCC. *CXCL5* (C-X-C Motif Chemokine Ligand 5) was shown to promote cancer cell proliferation [68]. *CYR61* (*CCN1*, cysteine rich angiogenic inducer 61) is involved in the induction of lipid metabolism and a metastatic phenotype in cancer cells [69]. *JUN* (Jun proto-oncogene, AP-1 transcription factor subunit) is involved in regulation of numerous cell activities, such as proliferation, apoptosis, survival, tumorigenesis, and tissue morphogenesis [70]. *THBS1* (thrombospondin 1) even contributes to the regulation of tumor cell adhesion, invasion, migration, and proliferation [71]. 

Three of the above mentioned genes have actually been closely linked to HCV-induced development of HCC. *AREG* is induced during acute and chronic liver injury, and it can protect liver cells from apoptosis. *AREG* is upregulated in hepatoma cells by HCV NS3 protein and stimulates hepatocarcinoma cell growth. Due to the activation of the cellular survival pathways, *AREG* may counteract HCV-induced apoptosis of infected hepatocytes and facilitate the development of liver cirrhosis and hepatocellular carcinoma. Moreover, *AREG* expression is essential for efficient HCV assembly and virion release [72]. 

*UAP1L1* (UDP-*N*-acetylglucosamine pyrophosphorylase 1 like 1) appears to be a regulator of O-GlcNAc transferase function and is a critical factor for cell proliferation in human hepatocarcinoma cells [73]. *UAP1L1* is significantly upregulated in a distinct subset of HCC tissues, and patients with upregulated expression of *UAP1L1* appeared to have a poor prognosis [73]. 

Also *SQSTM1* (p62, sequestosome) is upregulated in HCV replicating Huh-7.5 cells. *SQSTM1* is involved in regulation of autophagy and has been found to be upregulated in HCV-positive HCC patients [29]. *SQSTM1* was reported to promote malignancy through *Nrf2*-dependent metabolic reprogramming [29]. Thereby, enzymes of the pentose-phosphate pathway are upregulated, and the concentrations of glutathione and other metabolites involved in biotransformation and drug resistance increased, while the concentrations of metabolites of the glycolysis and the citric acid cycle were decreased. In conclusion, the upregulation of the above genes by HCV replication even in the hepatocarcinoma cell line Huh-7.5 indicates a further consolidation of the cancer state of these cells. 

### 2.5. Translational Upregulation of mRNAs with Upstream Open Reading Frames

One of the most well studied stress signaling pathways which influence global protein synthesis is phosphorylation of eIF2. It results in rapid depletion of TC (ternary complex eIF2*GTP*met-tRNAi) levels and ultimately leads to global translational repression [74]. Some cellular mRNAs, however, are preferentially translated under these conditions. It is believed that most of such mRNAs evade translational repression through utilization of specific uORFs in their 5′ leaders [37]. As HCV replication induces ER stress, it was shown that activation of eIF2 is phosphorylated in HCV infected hepatocarcinoma cells [22]. 

**Figure 4:**
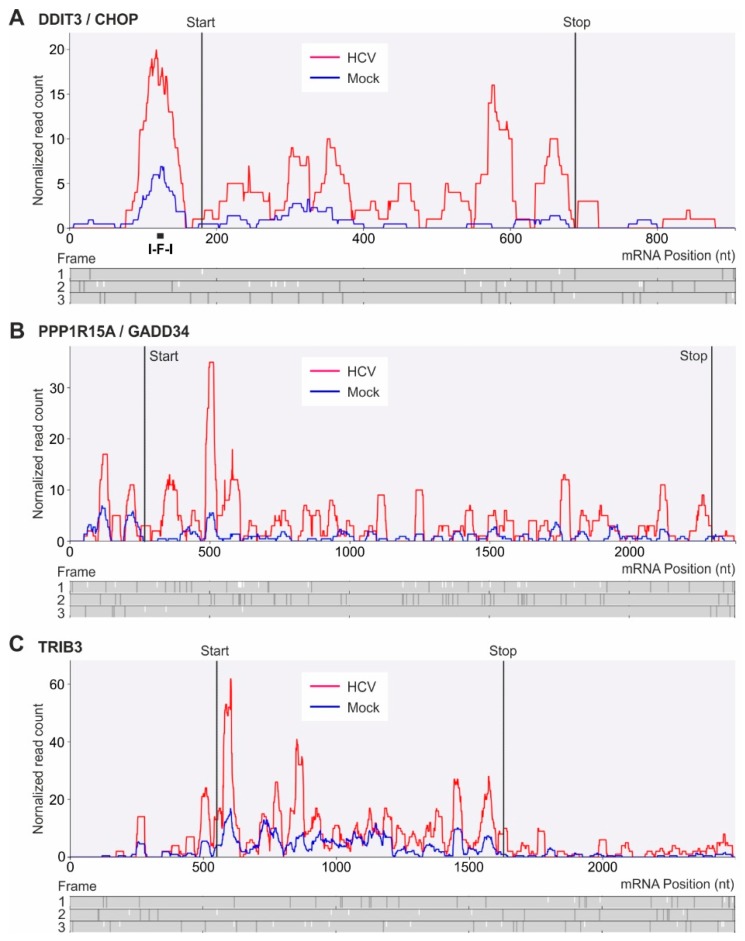
Detailed analysis of ribosome profiling read distribution of mRNAs regulated by upstream open reading frames (uORFs) during HCV replication, using Trips-Viz single transcript comparison plots [39]. (**A**) *DDIT3* (DNA Damage Inducible Transcript 3), also named *C*/*EBP Zeta* or C/EBP homologous protein (*CHOP*); Ensembl gene annotation of sliced transcripts: ENST00000346473. (**B**) *PPP1R15A* (protein phosphatase 1 regulatory subunit 15A), also called *GADD34* (Growth Arrest and DNA Damage-Inducible), ENST00000200453. (**C**) *TRIB3* (*C20orf97*, tribbles pseudokinase 3), ENST00000217233. Ribosome profiling read counts on mRNAs are shown in blue for mock-transfected and in red for HCV-transfected cells. Below the read profiles, reading frames counted from mRNA sequence entry nucleotide No. 1 are shown with start codons (small white bars) and stop codons (dark grey bars). A known ribosome stalling amino acid sequence (I-F-I) is indicated in (**A**).

Indeed, we observed that stress responsive *DDIT3* and *PPP1R15A* mRNA translation is upregulated (Figure 3C). Somewhat surprisingly, other mRNAs that are known to be differentially regulated upon ER stress, including the archetypical example *ATF4* mRNA, seem to be unchanged after 6 days of HCV replication, while translation of *TRIB3* largely escapes translational downregulation. These three mRNAs have translated upstream open reading frames (uORFs) in their 5′UTRs (Figure 4).

Stress conditions were reported to facilitate the ribosomal bypass of an inhibitory uORF in the *DDIT3* mRNA to enhance translation of the main ORF in the mouse *DDIT3* mRNA [75]. A later study from the same group reported that a ribosome stall in the uORF is involved in regulating *DDIT3* translation during the stress response. This stall is facilitated by the inhibitory amino acid sequence Ile-Phe-Ile (I-F-I) encoded in the uORF of the mouse *DDIT3* mRNA [76]. In the human *DDIT3* sequence analyzed here, we find a similar uORF arrangement (Figure 4A). The main *DDIT3* ORF is in frame 1. The uORF with two in-frame start codons is in frame 2. Also in the human *DDIT3* mRNA sequence, there is an I-F-I sequence four codons upstream of the uORF stop codon in frame 2. Exactly at this position, we find a large ribosome peak in the 5´UTR of the *DDIT3* mRNA (Figure 4A). We conclude that this inhibitory I-F-I sequence also plays a major role in translation regulation of human *DDIT3*/*CHOP* during stress response. 

During HCV replication, *DDIT3* is transcriptionally upregulated. Therefore, the ribosome coverage of *DDIT3* mRNA is higher due to increased mRNA abundance (red in Figure 4A). However, on top of this transcriptional upregulation during HCV replication, the ribosome coverage of the main ORF in relation to the uORF again increases by an additional factor of about 2. This suggests that the uORF with its ribosome stall site may be functionally involved in the upregulation of human *DDIT3* main frame translation during the stress response. Our results show that *DDIT3*/*CHOP* is activated during HCV-induced ER stress not only indirectly by other transcription factors [56] but also using uORF-mediated translational regulation which actually is conferred by ribosome stalling. 

In the *PPP1R15A* gene, uORFs regulate translation of the *PPP1R15A* main ORF during cellular stress [37,77]. In the mouse *PPP1R15A* gene, there are two uORFs in the *PPP1R15A* mRNA 5′UTR that overlap [78]. It was proposed for the mouse *PPP1R15A* mRNA that under normal conditions ribosome reinitiation on the second of the two overlapping uORFs directs translation of the second uORF and by that downregulates *PPP1R15A* main ORF translation. Under stress conditions, both uORFs are passed by scanning ribosomes which then start translation at the main ORF initiation codon [78,79]. In contrast, in the human *PPP1R15A* gene, the two uORFs are separated. Initiation of the second uORF was reported to be stronger than that of the first uORF, but both uORFs are required for efficient downregulation of *PPP1R15A* main ORF translation [77]. 

Our results (Figure 4B) indicate that both uORFs in the human *PPP1R15A* mRNA (frame 1) are occupied by ribosomes under normal conditions as well as under conditions of HCV replication, with a tendency for a higher ribosome occupancy of uORF1. However, under normal conditions occupation of the stop codon of uORF2 interferes with translation initiation at the main ORF initiation codon (in frame 3) which is too close to the uORF2 termination site. In contrast, under conditions of HCV replication, stalling of ribosomes in the uORFs results in higher efficiency of main ORF translation. When compared with normal conditions, under HCV replication conditions the ribosome coverage of the main ORF is more than doubled in relation to ribosome occupancy of the uORFs. Thus, we conclude that during HCV replication both transcriptional and translational upregulation of *PPP1R15A* contribute to *PPP1R15A* expression. 

*TRIB3* mRNA also largely escapes translational downregulation (see Figure 3A,C). *TRIB3* mRNA was reported to have an inhibitory uORF in the 5′UTR [66]. The main *TRIB3* ORF is in frame 2 (Figure 4C). Our inspection of the human *TRIB3* mRNA sequence (ENST00000217233) revealed a short uORF1 in frame 3 (position 129–155), followed by a second, longer uORF2 also in frame 3 (starting at position 189) with two potential stop codons (pos. 420 and 510). A strong ribosome peak at position 510 must be attributed to termination at the second uORF2 stop codon. However, a second, smaller ribosome peak at about position 260 in uORF2 (Figure 4C) could be speculated to be due to ribosome stalling in the uORF2 sequence, similar to the ribosome stalling sequence in the *DDIT3* uORF (see above). 

## 3. Conclusions

After establishment of HCV replication in the cells we observe upregulation of a limited number of stress-induced genes. The low number of regulated genes may in part be due to the fact that the use of Huh-7.5 hepatocarcinoma cells in this study comes with the limitation that these cells already had undergone changes in gene expression towards cancer. HCV-regulated genes include *PPP1R15A* which is counter-acting stress induction in order to normalize cellular gene expression. This may be important for HCV to further replicate “under the radar” in order to allow chronic infection without strong immune response. Our results also substantially add to the understanding of the downregulation of oxidative phosphorylation in tumor cells. Downregulation of the mRNA expression coding for key subunits of respiratory chain complexes I and IV in conjunction with the upregulation of *SESN2* and *SQSTM1* occurs rather early after establishment of HCV replication in the cells. The resulting metabolic changes in glycolysis and pentose phosphate pathway may further contribute to reprogramming the cells towards development of hepatocellular carcinoma and establishing the Warburg effect in the cancer cells.

## 4. Material and Methods

### 4.1. Cell Culture

Huh-7.5 cells [80] were maintained in Dulbecco’s modified Eagle medium supplemented with 10% fetal bovine serum (FBS), 100 µg/mL penicillin as well as 100 μg/mL streptomycin at 37 °C in 5% CO_2_. 

### 4.2. In Vitro Transcription of HCV-RNA

JFH1/J6 chimeric full-length HCV genome (JC1) [34] was generated by in vitro-transcription. Plasmids were first linearized with MluI-HF (NEB) and purified with phenol/chloroform extraction and precipitation by ethanol. Nucleic acid concentrations were measured using Qubit fluorimeter with corresponding assay kits. For transcription, 1 U/µL T7-RNA-Polymerase, 1 fold buffer, 3.75 mM of each rNTP, 10 mM DTT, 5 mM MgCl_2_ and 30 µg/mL DNA template was combined and incubated at 37 °C for 5 h. To remove the DNA template, 0.1 U/µL DNase I with corresponding buffer was added and incubated at 37 °C for 1 h. HCV-RNA was purified with “GeneJet RNA Cleanup and Concentration Micro Kit” (Thermo Scientific, Waltham, MA, USA) according to the manufacturer’s protocol. RNA integrity was confirmed by 0.8% agarose gel electrophoresis.

### 4.3. miR-122 Preparation

miR-122 oligonucleotides were obtained from Biomers. Mature miR duplexes were generated by hybridizing equimolar amounts of guide (5′-(phos)UGGAGUGUGACAAUGGUGUUUG-3′) and passenger strands in a thermocycler by slow temperature decrease from 90 to 4 °C (1 °C per minute). 

### 4.4. Transfection

Huh-7.5 cells were seeded (5 × 10^3^ cells/cm^2^) in 15 cm dishes and 12 well plates for ribosome profiling as well as for control experiments. One day after seeding, 0.3 pmol double-stranded miR-122/cm^2^ with or without 0.01 pmol HCV-RNA/cm^2^ were transfected with Lipofectamine 2000 (Invitrogen, Carlsbad, CA, USA). After 72 h, miR-122 duplexes were transfected again as before. Ectopic miR-122 was supplemented to elevate miR-122 in the HuH-7.5 cells which is not as high as in human primary liver cells [15,81]. Six days post transfection of HCV-RNA cells were lysed for qPCR, western blot and ribosome profiling.

### 4.5. RT-qPCR

To evaluate HCV-RNA production, RT-qPCR was performed as described before [82]. Briefly, cells were lysed with Trizol LS reagent (Ambion), and total RNA was extracted with chloroform and precipitated with isopropanol. After DNase I treatment and RNA isolation, cDNA was produced with the qScript Flex cDNA Kit (Quanta Biosciences, Beverly, MA, USA). qPCR was performed using PerfeCTa SYBR Green FastMix (Quanta Biosciences). Intracellular miR-122 amounts were quantified using the Taq-Man microRNA detection system (Thermo Scientific) for miRNA-122 as well as miRNA-22 assay (for normalization).

### 4.6. Western Blot

HCV protein translation was verified by western blot. Transfected Huh-7.5 cells were harvested with lysis buffer (10 mM Tris-HCl (pH 8), 140 mM NaCl, 0.025% NaN_3_, 1% Triton X-100) and incubated for 20 min at 4 °C. After centrifugation lysates were mixed with SDS sample buffer, denatured (90 °C) and loaded on a SDS-polyacrylamide gel. After blotting (0.8 mA/cm^2^), membranes were blocked (5% milk powder in PBS). HCV NS3 protein was detected with 8G-2 antibody (Abcam, 1:500), core protein with C7-50 antibody (Thermo Scientific, 1:1000) and cellular GAPDH with 71.1 antibody (1:20,000, Sigma-Aldrich, St. Louis, MO, USA). As secondary antibody a goat-anti-mouse IgG HOR (1:20,000, Life Technologies, Carlsbad, CA, USA) was used. Target proteins were detected with SuperSignal West Femto Chemiluminescent Substrate (Thermo Scientific).

### 4.7. Ribosome Profiling and RNA-Seq Library Preparation

Library preparation was performed as described in [36] with additional modifications listed below. Six days post transfection cells were washed with ice-cold PBS with cycloheximide (Sigma Aldrich, 100 µg/mL) and harvested by adding 500 µL polysome lysis buffer (20 mM Tris–HCl (pH 7.5), 250 mM NaCl, 1.5 mM MgCl_2_, 1 mM DTT, 0.5% Triton X-100, 100 μg/mL, 20 U/mL TURBO DNase) per 15 cm^2^ dish. Cells were not triturated with a gauge needle. After centrifugation, the supernatant was divided into two parts for ribosome profiling and for total RNA library preparation. In one part RNA was quantified and digested with RNase I (100 U/3 A260) at room temperature for 50 min. To stop the digest, 40 U SUPERase*In per 100 U of RNase I was added. Sucrose gradient ultracentrifugation was performed as described in [37] to extract and concentrate 80S ribosomal protected RNA fragments. Gradients were fractionated and nucleic acid content measured at 260 nm. Fractions from the 80S monosome peaks were combined, phenol/chloroform extracted and precipitated with isopropanol. For total RNA library preparation, at first Trizol LS reagent was used according to the manufacturer’s protocol. After ethanol precipitation, alkaline fragmentation was performed [35] for 60 min. From this point on, ribosome protected and fragmented total RNA samples were treated the same way. Samples were loaded on 15% polyacrylamide TBE-urea gels next to marker oligonucleotides, and fragments with a length of 26 to 34 nucleotides were excised. RNA was extracted from gel fragments by adding 1 mL RNA extraction buffer and incubating overnight at room temperature on a shaker. Library preparation was conducted as described before [36] with the following modifications. On the one hand, the circularization reaction was performed for two hours instead of one hour. For rRNA depletion, 14 µL sample reaction, 2 µL 20 × SSC, 2 µL water and 2 µL rRNA subtraction oligonucleotide pool (D.E.A.) was combined. Prior to actual PCR amplification with barcodes, optimal PCR cycle number was determined in 20 µL volume reactions. Flanking sequences of PCR products were as follows: 5′AATGATACGGCGACCACCGA GATCTACACTCTTTCCCTACACGACGCTCTTCCGATC (NEBNext Universal PCR Primer for Illumina)—Insert—ACTGTAGGCACCATCAAT (Adapter)—AGATCGGAAGAGCACAC GTCTGAACTCCAGTCACXXXXXXATCTCGTATGCCGTCTTCTGCTTG (NEBNext Index Primer for Illumina). Three replicates were carried out in parallel, RNA integrity verified on an Agilent BioAnalyzer and sent for sequencing on a HiSeq 4000 at BGI Genomics (Shenzhen, China). 

### 4.8. Preprocessing of Reads and Principal Component Analysis

To quickly assess the overall quality and usability of the sequencing data, a principal component analysis (PCA) was performed. We therefore performed an initial mapping and differential expression analysis (DEA).

DNA linker and subsequent Illumina Universal Adapter sequences were trimmed using cutadapt (version: 1.15) (parameters: -O 1 -a ACTGTAGGCACCATCAATAGATCGGAAGAG). Short reads and empty sequences were first removed using cutadapt (-m 25), and low quality reads were removed using fastq_quality_filter from the FASTX Toolkit (0.0.14) (-q 14 -p 96). The final window size was determined by cutadapt (-f fastq -q 28 -m 25 -M 34). Mapping of the remaining ribosome footprints against a human rRNA dataset removed ribosomal derived sequences. Bowtie2 (2.3.4) (--seedlen 20 --un) served for this filtering step, keeping only non-mapping reads. The human rRNA sequences were obtained from various sources, including Ensembl BioMart (Ensembl Genes 91; GRCh38.p10; filters: gene type: rRNA and Mt_rRNA), the SILVA rRNA database project [83] (with fasta headings “>id Homo sapiens (human)”) and four additional rRNA sequences obtained from the RiboGalaxy project [84]. Reads withstanding the filtering were subject to mapping against the human and HCV genomes using bowtie2 (2.3.4) (-N 1 -L 22 -p 8 --no-unal -x <human_and_hepC_genome_index>). The human genome sequence was obtained from Ensembl (Homo_sapiens.GRCh38.dna.primary_assembly.fa). Reads with a MAPQ score below 20 were discarded. Finally the remaining reads were converted into BAM format using samtools view (1.7) (-bS).

To generate a principal component analysis (PCA) we performed an initial differential expression analysis (DEA) using R (3.4.2) and the Bioconductor package DESeq2 (1.18.2) [85]. A gene model for the read counting step was derived from the human genome annotation in gtf format (Homo_sapiens.GRCh38.90.gtf) as obtained from Ensembl. To generate the actual read counting data, the two BioConductor packages GenomicAlignments (1.14.1) [86] and BiocParallel (1.12.0) were utilized, extracting the count matrix from the previously generated BAM files. Genes without any mapping reads were excluded from the analysis. Results as obtained from DESeq2’s plotPCA function were visualized using ggplot2 (3.0.0). 

### 4.9. Trips-Viz Preprocessing and Mapping of Sequencing Data

The Trips-Viz (“TRanscriptome wide Information on Protein Synthesis VIZualized”; http://trips.ucc.ie/) is a transcriptome browser designed to visualize Ribosome profiling and RNA-seq data at the level of a single gene/transcript isoform [39]. Pre-processing and mapping pipeline is as follows:

The adapter sequences were removed from reads using cutadapt [87] (version 1.18) with the following command: cutadapt -f fastq -a CTGTAGGCACCATCAAT --minimum-length=25 <input>.fastq -o <output>.fastq. 

Human rRNA sequences were retrieved from NCBI [88] with the following accessions: NR_003286, NR_003287, NR_023363, NR_003285. Reads aligning to these sequences were then removed using bowtie (version 1.0.1) with the following command: bowtie -v 3 --norc <path_to_rRNA_indices> -q <input>.fastq --un <output>.fastq. 

Bowtie [89] was then used to align the remaining reads to a transcriptome consisting of a concatenation of the GENCODE [90] version 25 human transcriptome and the HCV genomic sequence. The following command was used: bowtie --norc -a -m 100 -n 2 --seedlen 25 <path_to_transcriptome_indices> -q <input>.fastq <output>.sam. 

The SAM file was then converted to a BAM file and sorted by readname using samtools [91] (version 0.1.19) and the following commands: samtools view -b -S <input>.sam -o <output>.bam; samtools sort -m 1G -n <input>.bam <output>.bam_sorted. 

The genomic position(s) of each mapped read was determined, and if the read aligned to only one genomic position it was classed as an unambiguously mapped read, otherwise the read was classed as an ambiguously mapped read.

### 4.10. Differential Expression Analysis

For differential translation analysis using Trips-Viz [39], a principal transcript isoform was chosen for each gene locus from the APPRIS database [92] (http://appris.bioinfo.cnio.es/) for GENCODE version 25. The translation efficiency (TE) was calculated for each transcript by dividing Ribo-seq counts (within CDS) by RNA-Seq counts (entire transcript). Differential gene expression plots use the Z-score approach as described in [37] to determine which transcripts are up- or downregulated. The Z-score represents how many standard deviations from the mean a particular transcript is. However since it can be expected that more variance is in lowly expressed transcripts than in highly expressed ones, transcripts were first placed into groups of similar expression levels. The geometric mean of Ribo-Seq/RNA-Seq counts from control/infected samples was calculated for each transcript. Transcripts were then sorted from lowest to highest geometric mean and grouped into bins of 300. For each bin the TE fold change between control/infected samples was calculated and the mean and standard deviation of fold changes in each bin was used to calculate a Z-score for each transcript. 

Differential expression analyses were visualized by the Trips-Viz program package. Differential gene expression plots were generated for pairwise comparisons of each of the three replicates of one treatment (e.g., HCV) against each replicate of the other treatment (mock) in all possible combinations. 

### 4.11. Metagene Analysis

A metagene profile was created for each Ribo-Seq read length from each dataset, centered on the annotated start codons. The distance between the start codon and highest peak upstream of the start codon plus 3 nucleotides was used as an offset from the 5′ end of each read to infer the ribosome A-site. Metagene analyses were generated using the Trips-Viz transcriptome browser environment (http://trips.ucc.ie/).

### 4.12. Single Transcript Comparison Plots

Single transcript comparison plots were generated using Trips-Viz (http://trips.ucc.ie/). Reads were normalized by mapped reads, and windows were set to 25–40 nucleotides.

### 4.13. Deposition of Sequences and of Expression Data

Sequence and expression analysis information is available in the NCBI GEO database: https://www.ncbi.nlm.nih.gov/geo/query/acc.cgi?acc=GSE127713.
